# Improved high quality sand fly assemblies enabled by ultra low input long read sequencing

**DOI:** 10.1038/s41597-024-03628-y

**Published:** 2024-08-24

**Authors:** Michelle Huang, Sarah Kingan, Douglas Shoue, Oanh Nguyen, Lutz Froenicke, Brendan Galvin, Christine Lambert, Ruqayya Khan, Chirag Maheshwari, David Weisz, Gareth Maslen, Helen Davison, Erez Lieberman Aiden, Jonas Korlach, Olga Dudchenko, Mary Ann McDowell, Stephen Richards

**Affiliations:** 1 Department of Biological Sciences, University of Notre Dame, Notre Dame, IN USA; 2 Pacific Biosciences, Menlo Park, CA USA; 3 DNA Technologies and Expression Analysis Cores, UC Davis Genome Center, University of California, Davis, Davis, CA USA; 4 The Center for Genome Architecture, Baylor College of Medicine, Houston, TX 77030 USA; 5 Department of Molecular and Human Genetics, Baylor College of Medicine, Houston, TX 77030 USA; 6 Department of Life Sciences, Imperial College London, South Kensington Campus, London, SW7 2AZ UK; 7 Institute of Systems, Molecular and Integrative Biology, University of Liverpool, Liverpool, UK; 8 Center for Theoretical and Biological Physics, Rice University, Houston, TX 77030 USA; 9 Broad Institute of Harvard and Massachusetts Institute of Technology (MIT), Cambridge, MA 02139 USA; 10 Eck Institute for Global Health, University of Notre dame, Notre Dame, IN USA; 11 Human Genome Sequencing Center, Baylor College of Medicine, Houston, Texas USA

**Keywords:** Comparative genomics, Genomics, Parasitic infection, Genome assembly algorithms

## Abstract

Phlebotomine sand flies are the vectors of leishmaniasis, a neglected tropical disease. High-quality reference genomes are an important tool for understanding the biology and eco-evolutionary dynamics underpinning disease epidemiology. Previous leishmaniasis vector reference sequences were limited by sequencing technologies available at the time and inadequate for high-resolution genomic inquiry. Here, we present updated reference assemblies of two sand flies, *Phlebotomus papatasi* and *Lutzomyia longipalpis*. These chromosome-level assemblies were generated using an ultra-low input library protocol, PacBio HiFi long reads, and Hi-C technology. The new *P. papatasi* reference has a final assembly span of 351.6 Mb and contig and scaffold N50s of 926 kb and 111.8 Mb, respectively. The new *Lu. longipalpis* reference has a final assembly span of 147.8 Mb and contig and scaffold N50s of 1.09 Mb and 40.6 Mb, respectively. Benchmarking Universal Single-Copy Orthologue (BUSCO) assessments indicated 94.5% and 95.6% complete single copy insecta orthologs for *P. papatasi* and *Lu. longipalpis*. These improved assemblies will serve as an invaluable resource for future genomic work on phlebotomine sandflies.

## Background & Summary

Phlebotomine sand flies (family Psychodidae, order Diptera) include several genera of hematophagous arthropods that vector important emerging and re-emerging infectious diseases. They transmit bacterial, viral, and, most notably, the protozoan pathogen *Leishmania*, to humans and animals. Leishmaniasis is a group of diseases that range in clinical manifestation, from self-healing cutaneous lesions to disfiguring mucocutaneous ulcers to fatal visceral disease. Clinical tropisms can be highly dependent on infective species and vectoring sand fly. Over 90 species of sand flies found across Latin America, Africa, the eastern Mediterranean, Southeast Asia, and Europe have been implicated as vectors for approximately 20 species of *Leishmania* parasites that cause leishmaniasis^1,2^.

*Phlebotomus papatasi* vectors *Leishmania major*, an etiological agent of cutaneous leishmaniasis, across North Africa, the Middle East, and the Indian subcontinent^3^. It is a restrictive vector in that it can only transmit a single *Leishmania* species, *Le. major*. However, *P. papatasi* also transmits viral febrile illnesses across its distribution^4,5^. *Lutzomyia longipalpis* is the major vector responsible for transmission of the visceral leishmaniasis causing parasite, *Leishmania infantum*, in the Americas^6^. *Lu. longipalpis* is a permissive vector in the laboratory, transmitting several *Leishmania* species, however in nature it only transmits *Le. infantum*^7^. *Lu. longipalpis* has a wide geographic distribution inhabiting a range of diverse ecological habitats and has garnered interest as a species complex. Others have observed differences in spot numbers, pheromones, mating songs, and noted reproductive isolation between different populations collected throughout Brazil^8^. Leishmaniasis pathogenesis is thought to be dependent on complex host, vector, and parasite interactions and, although the epidemiological implications of a *Lu. longipalpis* species complex remain unclear, understanding the molecular underpinnings that that lead to vector competence, reproductive isolation and adaptation is critical from an epidemiological and disease control perspective.

In mosquito research, high-quality reference genomes have enabled inquiries into population genetics and metagenomics, identification of gene markers of senescence, vector competence, insecticide resistance, and experimental gene drive approaches to vector control. These have ultimately improved understanding and management of the vector in the disease transmission cycle^9^. Unfortunately, the fragmented nature of current sand fly references slowed similar inquiries for *Leishmania* transmission.

Previous reference genomes for *P. papatasi* and *Lu. longipalpis*^10^ suffered very low contiguity. Using the best sequencing technology at the time, read lengths were limited to ~400 bp - too short to span many repeats. More damaging to assembly contiguity, previous library protocol DNA input minimums required DNA to be pooled from many individuals, inserting many different haplotypes into the assembly algorithm. Genome heterozygosity could not be controlled for by inbreeding in sand flies, and haplotype sequence variation – for example, a short insertion polymorphism – caused assembly tools designed for a single haplotype to create sequence gaps in areas of uncertainty. Together, these constraints led the genome assemblies for *P. papatasi* and *Lu. longipalpis* to be the 2^nd^ and 3^rd^ worst available in VectorBase^11^, with contig N50 lengths at 5,795 bp and 7,481 bp, respectively. For reference, across all genomes in VectorBase at the time, the median assembly contig N50 was 51,691 bp. Additionally, no Hi-C or chromosome scale data was available, and these fragmented genome assemblies were inadequate for many genome analyses.

Here, we update these two important sand fly vector genome references leveraging a decade’s worth of technological advances. Specifically, very high quality long read sequences of Q20 or even Q30 are available in lengths longer than the previous assemblies contigs. Second, Hi-C technologies have become de rigueur and have higher chromosomal completion rates when paired with the significantly longer contigs generated by high quality long read assembly. Finally, an ultra-low input library protocol developed by Pacific Biosciences^12^ enabled the sequencing of a single individual sand fly. This greatly simplified assembly of sequence information from only 2 haplotypes derived from a single individual rather than many haplotypes from a pool of individuals. A small compromise, as only 30 ng of genomic DNA can be isolated from a single sand fly male, is the use of whole genome amplification. Together these three techniques have generated the greatly improved reference assemblies we describe here.

## Genome Sequence Report

The genomes of *P. papatasi* and *Lu. Longipalpis* were each sequenced from a single male from colonies maintained at the University of Notre Dame. The *P. papatasi* colony was established in the 1970s from the Israeli strain and the *Lu. Longipalpis* colony was established in 1988 from the Jacobina strain caught from Bahia State, Brazil. *P. papatasi* sequencing generated 102x coverage and *Lu. longipalpis* sequencing generated 53x coverage of PacBio HiFi long reads. Additional material from other individuals from the same colonies was used for Hi-C library preparation.

**Fig. 1:**
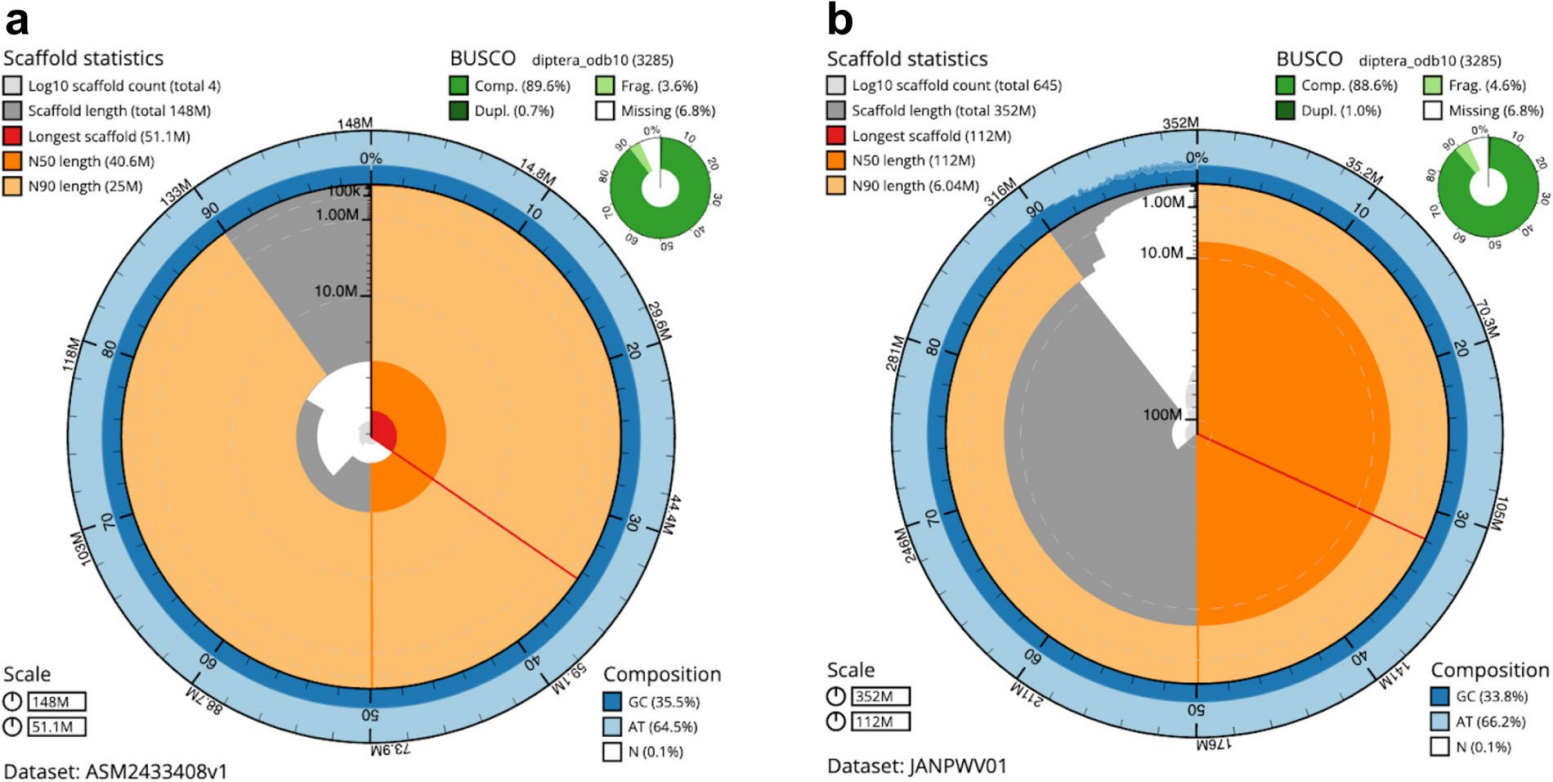
Snail plot summaries of assembly statistics. (**a**) *Lutzomyia longipalpis* assembly ASM2433408v1. (**b**) *Phlebotomus papatasi* assembly JANPWV01. Both plots were generated using blobtoolkit^43^.

**Fig. 2:**
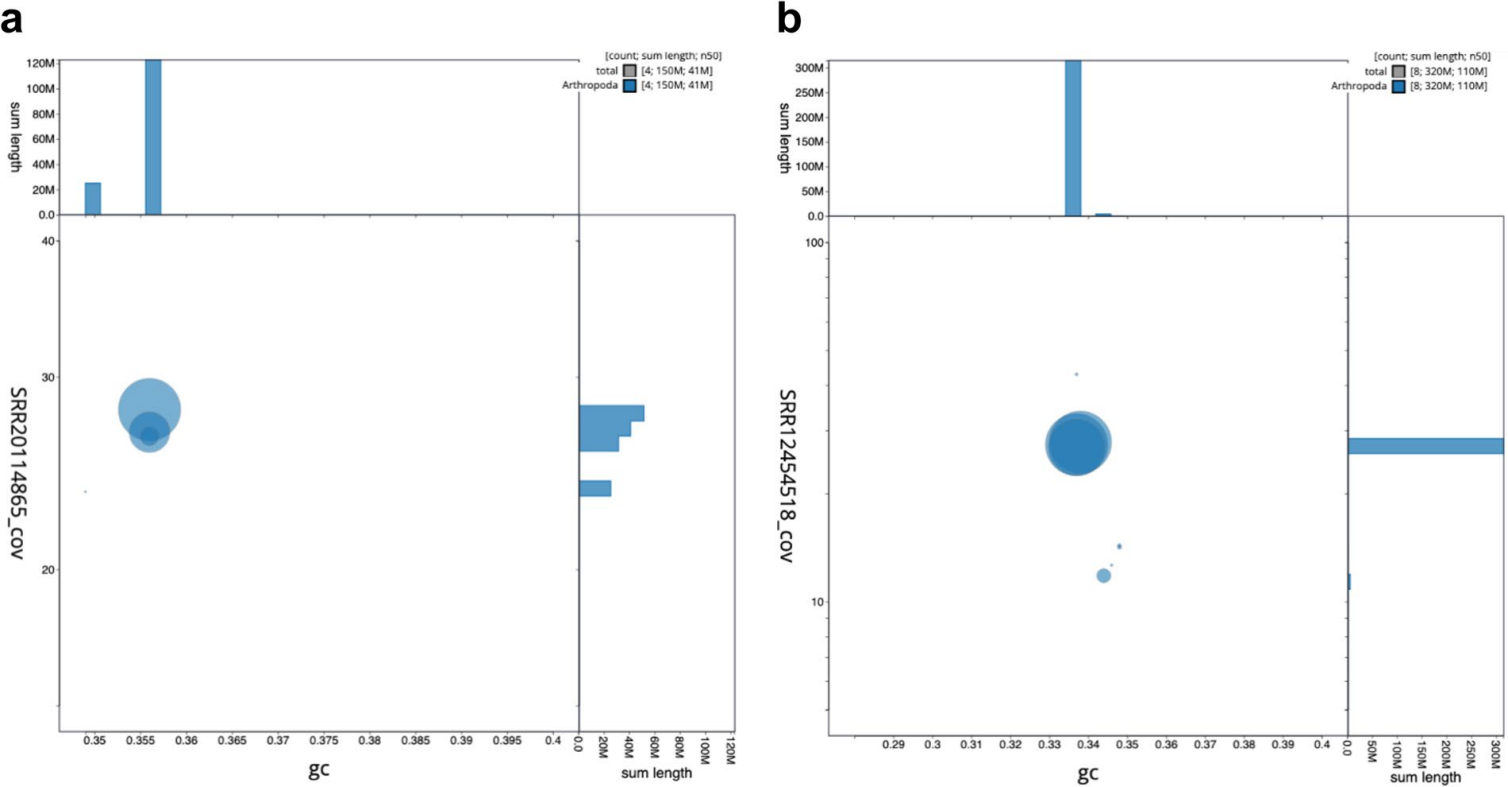
Blobplots of base coverage against GC proportion. (**a**) *Lutzomyia longipalpis* assembly ASM2433408v1. (**b**) *Phlebotomus papatasi* assembly JANPWV01 with no-hits filtered out. Both plots were generated using blobtoolkit^43^.

The final *P. papatasi* assembly has a span of 351.6 Mb, 646 scaffolds, and a scaffold N50 of 111.8 Mb. The final *Lu. Longipalpis* assembly has a span of 147.8 Mb, 4 scaffolds, and a scaffold N50 of 40.6 Mb (Table 1, Figs. 1 & 2). The updated assemblies improved upon several deficiencies from the previous assemblies (Table 2). Compared to the previous assemblies, contiguity has improved over 100-fold and these larger contigs are placed in a chromosomal context.

**Table 1:** Genome data and global statistics.

	*Phlebotomus papatasi*	*Lutzomyia longipalpis*
Project accession data		
Assembly identifier	Ppap_2.1	ASM2433408v1
Specimen	Single male, Notre Dame Colony, Israeli Strain	Single male, Notre Dame Colony, Jacobina Strain
NCBI taxonomy ID	29031	7200
BioProject	PRJNA858452^36^	PRJNA849274^30^
BioSample ID	SAMN15793614	SAMN29048364
Isolate information	M1	SR_M1_2022
SRA long reads	SRX8948934^33^	SRX16150135^28^
SRA Hi-C reads	SRX18440491^37^	SRX18440490^31^
Genome assembly		
GenBank accession	GCA_024763615.2^34^	GCA_024334085.1^29^
RefSeq accession	GCF_024763615.1	GCF_024334085.1
Sequence length	351,623,088	147,838,017
Number of contigs	1,350	255
Contig N50 length	926,603	1,092,454
Number of scaffolds	646	4
Scaffold N50 length	111,783,093	40,620,313
# chromosomes	6	4

**Table 2:** Comparison of old and new assembly statistics.

	*P. papatasi*		*Lu. longipalpis*	
	Old	New	Old	New
Genome Size	363,767,908 bp	351,623,088 bp	154,229,266 bp	147,838,017 bp
Coverage	15.1x	113.5x	38.9x	53x
Contig N50	5.8 kb	926.6 kb	7.5 kb	1,092.5 kb
Contig Count	139,199	1,349	35,969	255
Scaffold N50	27,956 bp	111.8 Mbp	85,093 bp	40.6 Mbp
Scaffold Count	106,826	645	11, 532	4
Coding Genes	11,377	11,610	10,422	11,236
Noncoding Genes	444	995	338	778
BUSCO	86.5%	95.2%	86.1%	96.6%
NCBI Accession #	GCA_000262795.1	GCF_024763615.1	GCA_000265325.1	GCF_024334085.1
VectorBase	Past	Current Reference	Past	Current Reference

Two genome annotations are available for each species. The first is a new NCBI RefSeq^13^ annotation based on not just this assembly but also new long read transcript data generated to support new annotation. Gene numbers derived from this annotation are shown in Table 2 and BUSCO analysis in Table 3. The number of complete single copy insecta single copy orthologs increased by ~10%. That is, an additional 10% of genes that were previously incomplete or missing are now easily accessible in the improved assembly. In addition to this updated annotation resource, we wished to preserve previous annotations, especially user contributed curated annotations, which connect the genome to previously published analyses. To preserve previous annotation information, we utilized the new open-source pipeline *Transfer-annotations*^14^ developed by VectorBase engineers to iteratively run *Liftoff*^15^ to accurately transfer previous annotations to new VectorBase Apollo browser tracks and generate a downloadable GFF3 annotation file for each species.

**Table 3:** BUSCO results for two new sandfly references.

Reference	Dataset	Buscos	Complete	Duplicated	Fragmented	Missing
*P. papatasi*	diptera_odb10	3,285	2,910 (88.6%)	32 (1.0%)	150 (4.6%)	225 (6.8%)
	endopterygota_odb10	2,124	1,968 (92.7%)	26 (1.2%)	63 (3.0%)	93 (4.4%)
	insecta_odb10	1,367	1,301 (95.2%)	20 (1.5%)	24 (1.8%)	42 (3.1%)
*Lu. longipalpis*	diptera_odb10	3,285	2,943 (89.6%)	22 (0.7%)	117 (3.6%)	225 (6.8%)
	endopterygota_odb10	2,124	2,006 (94.4%)	11 (0.5%)	45 (2.1%)	73 (3.4%)
	insecta_odb10	1,367	1,320 (96.6%)	6 (0.4%)	18 (1.3%)	29 (2.9%)

## Methods

### Sample acquisition and nucleic acid extraction

Single males were chosen for sequencing to capture the heterogametic sex chromosomes, and to ensure only high quality long read sequence data from a single diploid genome was presented to the assembly software for facile assembly. A single male adult sand fly was aspirated from each of our *P. papatasi* and *Lu. Longipalpis* colonies and frozen at −80 °C. Each specimen was chilled in liquid nitrogen and ground into a fine powder preceding DNA extraction using a modified Puregene® kit extraction protocol (Qiagen, Hilden, Germany). DNA was eluted in 30 μl of TE buffer and concentration was assessed using a Nanodrop Spectrophotometer.

### Long read library construction and sequencing

Pacific Biosciences HiFi Libraries were constructed using an ultra-low input library protocol^12^. The *P. papatasi* library was prepared at Pacific Biosciences using a pre-production version of the library kit. The *Lu. Longipalpis* library was prepared at the UC Davis DNA technologies core using the commercially available SMRTbell gDNA Sample Amplification Kit (Pacific Biosciences, Menlo Park, CA; Cat. #101-980-000) and the SMRTbell Express Template Prep Kit 2.0 (Pacific Biosciences; Cat. #100-938-900) according to the manufacturer’s instructions. Briefly, approximately 10 kb sheared DNA by the Megaruptor 3 system (Diagenode, Belgium; Cat. #B06010003) was used for removal of single-strand overhangs at 37 °C for 15 minutes, DNA damage repair at 37 °C for 30 minutes, end repair and A-tailing at 20 °C for 30 minutes and 65 °C for 30 minutes, and ligation of overhang adapters at 20 °C for 60 minutes. To prepare for library amplification by PCR, the library was purified with ProNex beads (Promega, Madison, WI; Cat. # NG2002) for two PCR amplification conditions at 15 cycles each then another ProNex beads purification. Purified amplified DNA from both reactions were pooled in equal mass quantities for another round of enzymatic steps that included DNA repair, end repair/A-tailing, overhang adapter ligation, and purification with ProNex Beads. The PippinHT system (Sage Science, Beverly, MA; Cat # HPE7510) was used for SMRTbell library size selection to remove fragments <6–10 kb. The 10-11 kb average HiFi SMRTbell library was sequenced using one 8 M SMRT cell, Sequel IIe sequencing chemistry 2.0, and 30-hour movies each on a PacBio Sequel II sequencer.

### Long read assembly

The draft *Lu. longipalpis* genome assembly was assembled using hifiasm^16^ from HiFi data generated from a single male individual at the UC Davis Genome Core using the ultra-low input protocol. Filtering input reads to have an average quality >Q30 was found to give a more contiguous final assembly for this dataset than Q20 filtered reads and was used for the final assembly. The draft genome assembly for *P. papatasi* was generated at Pacific Biosciences based on HiFi reads generated at Pacific Biosciences with a library made from a single adult male individual using an ultra-low input library kit. The long-read assembly was performed using HGAP and Falcon^17^.

### 3D sequencing and assembly

**Fig. 3:**
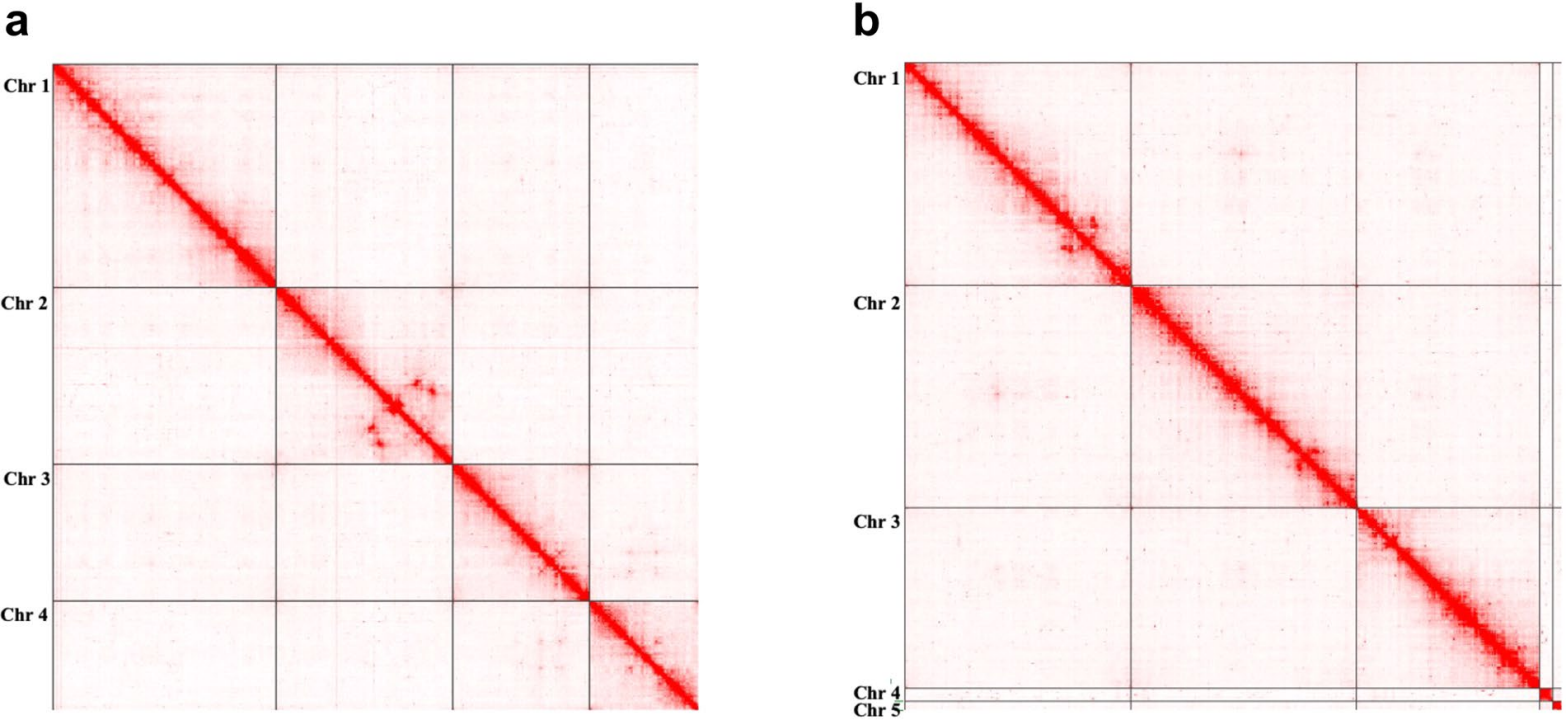
Hi-C contact maps. (**a**) *Lutzomyia longipalpis* (**b**) *Phlebotomus papatasi*. Chromosome-length Hi-C contact maps visualized in Juicebox^44^.

The high-quality drafts were upgraded to chromosome-length using Hi-C data derived from different male individuals from the same respective colonies at the University of Notre Dame. The *in situ* Hi-C libraries were generated as described in Rao, Huntley *et al*.^18^. Briefly, whole insect bodies were crosslinked with 1% formaldehyde for 10 minutes at room temperature. Nuclei were extracted via grinding and permeabilized using SDS. DNA was digested with a cocktail of Csp6I and MseI, and the ends of restriction fragments were labeled using biotinylated nucleotides then ligated. After reversal of crosslinks, ligated DNA was purified and sheared to a length of ~400 bp, at which point ligation junctions were pulled down with streptavidin beads and prepped for Illumina sequencing. The resulting libraries were sequenced using Illumina NovaSeq 6000 instruments. Hi-C data were aligned to the draft references using Juicer^19^, and 3D assembly for both species was performed using 3D-DNA pipeline^20^. In view of the large number of alternative haplotypes incorporated in the draft assembly as separate sequences^21^, 3D-DNA pipeline was run with the “merge” step option for *Lu. longipalpis* (see Matthews *et al*.^22^) to remove alt haplotypes from the anchored portion of the assembly. The resulting assemblies were reviewed and curated using Juicebox Assembly Tools^23^. The resulting contact maps (Fig. 3) can be explored interactively at multiple resolutions via Juicebox.js^24^ at the DNA Zoo website pages^25,26^.

### Removal of non-chromosomal sequences from *Lu. longipalpis*

During BUSCO analysis the *Lu. longipalpis* draft assembly contained high numbers of duplicate BUSCO genes. This was due to the presence of alternative haplotype sequences in the unanchored portion of the assemblies. As expected, removing unanchored sequences during annotation greatly reduced the duplicates.

### Gene annotation lift-over

We used the pipeline *Transfer-Annotations*^14^ and the program *Liftoff*^15^ to move previous gene annotations and manual curations to the new reference assembly. Liftoff distance and flank parameters were determined by incrementally changing them to find the combination with the lowest flank number and the fewest missing features. We used *agat_sp_fix_cds_phases*^27^ to calculate phase information and identify any transferred gene models that are incomplete or altered. AGAT’s *agat_sp_extract_sequences*^27^ was used to extract CDS protein sequences for the transferred genes on the new genome. The *Transfer-annotations* pipeline then identifies missing CDS regions, and it produces a corrected GFF3 with metadata regarding model validity in the GFF3 attributes column. This process includes if a protein sequence contains stop codons, if it matches the original sequence, or if it has any missing CDS regions. Transfers were considered invalid if the coding sequence had a missing CDS region or internal stop codon, or ncRNA sequences did not match between the source and transfer sequences. Coding sequences with mismatched protein sequences were not considered invalid and are flagged for future examination.

A final GFF3 of the transferred annotation is available at VectorBase as an Apollo genome browser track color coded by estimated transfer quality. A majority of genes transferred from each original source genome to its replacement assembly (Table 4). However, 30.3% and 22.0% were invalidated by missing CDS regions and internal stop codons, and 73.2% and 62.8% of CDS had mismatched protein sequences. That not all annotations could be transferred is likely unavoidable due to the differences in genome quality.

**Table 4:** Transfer summaries for *Lu longipalpis* and *P. papatasi*.

	*Lutzomyia longipalpis*	*Phlebotomus papatasi*
Source genome	*Lu. longipalpis* Jacobina, LlonJ1.6	*P. papatasi* Israel, PpapI1.6
Source accession	GCA_000265325.1	GCA_000262795.1
New genome	*Lu. longipalpis* M1, SR_M1_2022	*P. papatasi* M1, Ppap_2.1
New Accession	GCA_024334085.1	GCF_024763615.1
mRNA transcripts transferred	9,738 of 10,458 **(93.1%)**	11,070 of 11,405 **(97.1%)**
ncRNA transcripts transferred	276 of 338 **(81.7%)**	392 of 444 **(88.3%)**
Total transferred	10,014 of 10,796 (**92.8%)**	11,462 of 11,849 **(96.7%)**
Total invalid transfers	3,032 of 10,014 **(30.3%)**	2,516 of 11,462 **(22.0%)**
Total CDS with mismatched proteins	7,127 of 9,738 **(73.2%)**	6,956 of 11,070 **(62.8%)**

## Data Records

*Lutzomyia longipalpis* PacBio HiFi^28^ long reads and final assembly^29^ are available at the NCBI with BioProject accession number PRJNA849274^30^. *Lutzomyia longipalpis* HiC short reads are available at the NCBI SRA^31^ with BioProject accession number PRJNA512907^32^. *Phlebotomus papatasi* PacBio HiFi long reads^33^ and final assembly^34^ are available at the NCBI with BioProject accession numbers PRJNA657245^35^ and PRJNA858452^36^ respectively. *Phlebotomus papatasi* HiC short reads are available at the NCBI SRA^37^ with BioProject accession number PRJNA512907^32^. Additional sub-accessions are shown in Table 1.

## Technical Validation

One of our aims was for these new genome references to meet the Earth BioGenome Project standards^38^ despite the small amounts of input materials. Specifically, we aimed to have >1 Mb contig N50, and achieved full chromosome lengths using Hi-C data.

We assessed reference gene model completeness using BUSCO^39^ (V3.0.2). For both sandfly references the diptera_odb10 set of 2,910 single copy orthologs are missing 225 (6.8%) of the genes (Table 3). This number decreases when the analysis is performed on larger taxonomic groups with smaller BUSCO gene sets. For example, only ~3% of genes (*P. papatasi* (42) and *Lu. longipalpis* (29)) are missing from the 1,367 insecta_odb10 BUSCO gene set. Whilst this is a vast improvement on the previous assemblies, future work is required to determine which missing genes are due to assembly problems such as gaps between 1 Mb N50 contigs or genuine gene loss during >150 million years of divergence time between these species and others in the orthoDB database at the current time^40,41^.

While assessing base coverage and GC content for *P. papatasi*, we noticed a blob that stood out from the rest of the Arthropoda hits, with several-fold less base coverage (accession #: CM045756.1). Hits for this “blob” included families Culicidae, Curculionidae, formicidae, Kalotermitidae, Noctuidae, and Drosophilidae. To assess for contamination, we blasted these regions against the NCBI nucleotide database. The top hits returned *P. papatasi*. To investigate the possibility of a sex chromosome, we blasted Y chromosome-linked scaffolds in *Lu. longipalpis* identified by Vigoder *et al*. against the NCBI nucleotide database^42^. While there were several *P. papatasi* hits, none were localized to this blob. Interestingly, other hits included the X chromosome for several different species of flies, three of which have an XY sex chromosome system. Finally, we blasted our blob of interest against the *Drosophila* Y chromosome (NC_024512.1). There was no significant similarity found.

## Data Availability

No custom code was used to generate these assemblies. Long read assembly was performed hifiasm^16^, HGAP and Falcon^17^. Hi-C chromosomal scale assembly was performed using the Juicer/3D-DNA/Juicebox Assembly Tools pipeline^19,20,23^. For gene content analysis we used BUSCO version 3^39^. “Transfer-Annotations”, the code used to lift over previous curations to the new assembly is available on github^14^. This pipeline makes use of the tool Liftoff^15^.
